# Health Care Costs Associated with the Development and Combination of Cardio-Renal-Metabolic Diseases

**DOI:** 10.34067/KID.0000000000000212

**Published:** 2023-07-18

**Authors:** Gregory A. Nichols, Efrat L. Amitay, Satabdi Chatterjee, Dominik Steubl

**Affiliations:** 1 Kaiser Permanente Center for Health Research, Portland, Oregon; 2 Boehringer Ingelheim International GmbH, Ingelheim, Germany; 3 Boehringer Ingelheim Pharmaceuticals Inc., Ridgefield, Connecticut; 4 Department of Nephrology, Klinikum rechts der Isar, Technical University, Munich, Germany

**Keywords:** CKD, medical costs, type 2 diabetes, heart failure, atherosclerotic cardiovascular disease

## Abstract

**Key Points:**

- Onset of any new cardio-renal-metabolic condition drove substantial increase in health care costs.
- Overall costs increased by $10,316 (130%) when CKD developed, $6789 (84%) for type 2 diabetes, $21,573 (304%) for atherosclerotic cardiovascular disease, and $36,522 (475%) for heart failure.
- However, as a result of prediagnosis costs being higher as more conditions were present, the percentage increases in costs associated with incidence were lower when more prevalent conditions existed.

**Background:**

The cardio-renal-metabolic (CRM) syndrome is a constellation of conditions which includes atherosclerotic cardiovascular disease, heart failure (HF), CKD, and type 2 diabetes. The economic consequences of developing each of these comorbidities in the context of the others have not been studied.

**Methods:**

We used the electronic medical records of Kaiser Permanente Northwest to identify 387,985 members aged 18 years or older who had a serum creatinine measured between 2005 and 2017. Patients were followed through 2019. We used a statistical approach that assesses time dependency for continuous measures; the total observation period for each patient was divided into quarters (91-day increments), and each patient contributed a record for every quarter in which they were members of the health plan. CRM status was determined for each quarter.

**Results:**

The incremental annualized cost of each of these chronic diseases was similar regardless of which other conditions were present when the new condition developed. Overall costs increased by $10,316 (130%) when CKD developed, $6789 (84%) for type 2 diabetes, $21,573 (304%) for atherosclerotic cardiovascular disease, and $36,522 (475%) for HF. However, as a result of prediagnosis costs being higher as more conditions were present, the percentage increases in costs associated with incidence were lower when more prevalent conditions existed.

**Conclusions:**

Onset of any new CRM condition drove substantial increase in health care costs. Our findings indicate a clear interplay of CRM conditions and emphasize the need for better simultaneous prevention and management of these disease states to reduce the economic burden on health care systems.

## Graphical abstract

**Figure:**
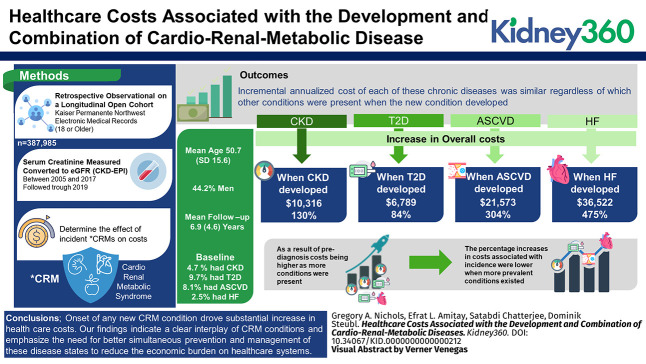

## Introduction

The cardio-renal-metabolic (CRM) syndrome is a constellation of conditions which includes atherosclerotic cardiovascular disease (ASCVD), heart failure (HF), CKD, and type 2 diabetes (T2D).^1^ It is well known that these diseases are interrelated^2–4^ and that they each escalate health care costs. For example, diabetes, most of which is T2D, accounts for one in every four health care dollars in the United States with medical expenditures, more than half of which is directly attributable to diabetes, which are 2.3 times higher than what they would be in the absence of diabetes.^5^ Medical costs associated with cardiovascular diseases (CVDs) are similarly high in the United States, exceeding $300 billion per year,^6^ while patients with HF incur annual costs that are nearly 4 times greater than patients without HF^7^;costs among elderly patients with CKD are at least double than those without CKD.^8^ Moreover, CRM conditions interact to escalate health care costs.^9^

Despite these known individual effects of prevalent CRM conditions, the economic consequences of developing each of these comorbidities in the context of the others have not been studied. An understanding of the long-term economic burden of the incidence of CRM components and their interplay could help decision makers allocate sufficient resources for CRM prevention and treatment. We undertook the current study to determine how costs change when ASCVD, HF, CKD, or T2D develop in the presence and absence of other conditions.

## Methods

We conducted a retrospective observational study of continuous outcomes on a longitudinal open cohort using the electronic medical records (EMRs) of Kaiser Permanente Northwest (KPNW), an integrated delivery system serving approximately 650,000 individuals in a 100-mile radius around Portland, Oregon. The KPNW EMR includes comprehensive databases of inpatient and outpatient encounters diagnoses, laboratory tests and results, and pharmaceutical dispenses. As a closed integrated delivery system, members receive essentially all of their medical care from KPNW clinicians and facilities, thus allowing for a complete assessment of medical costs. Because KPNW enrolls people whose insurance is self-paid, provided through employment, or by Medicare or Medicaid, the membership is representative of the service area.^10^ The KPNW institutional review board approved the study with a waiver of informed consent.

The study sample included all adults (older than 18 years) with at least one serum creatinine measurement between January 2005 and December 2017 (*N*=387,985). CKD was identified using serum creatinine measures converted to eGFR using the CKD Epidemiology Collaboration equation with race adjustment.^11^ CKD was considered present when an eGFR <60 ml/min per 1.73 m^2^ was recorded and confirmed by a second eGFR <60 within 3–12 months. If the second eGFR was not confirmatory, the patient was considered not to have CKD. If there was no second eGFR available within 12 months, we ignored the first measure and searched for the next available eGFR. If that measure was <60, we again looked for a confirmatory measure within 3–12 months. We repeated this process until an eGFR was >60 or until two measures within 3–12 months were <60. Once confirmed, the date of the first of the two eGFRs was considered the CKD diagnosis date and was used as the baseline date. For patients without CKD, we used the first available eGFR as the baseline date. We then used all available encounter data before the baseline date to determine whether T2D (International Classification of Diseases [ICD]-9: 250/ICD-10: E10, E11, E13), ASCVD (ICD-9: 410, 411, 412, 413, 414, 433, 434, 435/ICD-10: I20, I21, I22, I25, G45, I63, I65, I66), and HF (ICD-9: 428/ICD-10: I50) were present, defining data up to and including the baseline date as the baseline period.

The potential follow-up period extended from the baseline date until December 31, 2019, or until a patient died or left the health plan. New-onset CKD was identified from laboratory values as described above. We used the same ICD codes listed above to identify new onset of each of the other CRM diseases among those without each condition at baseline. We used the 12-month period before the baseline date to identify all other baseline data including demographics (age, sex, race/ethnicity), clinical risk factors (smoking status, BP, body mass index), and use of selected medications (angiotensin-converting enzyme inhibitors or angiotensin receptor blockers, statins, antihyperglycemics).

Our costing method was developed and validated for research and risk adjustment purposes by the KPNW Center for Health Research.^10^ For outpatient costs, this method creates standard costs for office visits by specialty/department and type of clinician (medical doctor versus Physician Assistant/Nurse Practitioner). The number of visits per department per clinician type is then multiplied by the appropriate unit cost. Pharmaceutical costs are calculated on the basis of retail prices. Inpatient costs are based on assigned diagnosis-related group codes for the primary reason for hospitalization. The average daily cost per diagnosis-related group is then multiplied by the length of stay. Costs for medical services incurred at facilities not owned by KPNW are based on the amount paid by KPNW to the nonplan provider. These methods ensure that although the costs reported may be specific to KPNW, they approximate the charges a nonmember would be billed if these same services were purchased from KPNW. All costs were adjusted to 2020 US dollars using the medical or pharmaceutical component of the Consumer Price Index.

Because we sought to determine the effect of incident CRMs on costs, we used a statistical approach that assesses time dependency for continuous measures (such as costs).^12,13^ In this method, the total observation period for each patient was divided into quarters (91-day increments), and each patient contributed a record for every quarter in which they were members of the health plan. CRM status was determined for each quarter. For example, in the analysis of costs before and after incident CKD, we used those without CKD at baseline and determined each of the other CRM conditions at baseline. A patient with no CRM conditions was assigned to the analysis category of no T2D, ASCVD or HF and followed them until a CRM condition developed. If, in this example, that condition was CKD (the outcome), costs for all quarters before CKD were included as prior costs, and costs for all quarters after CKD incidence were summed and annualized as after-CKD costs. However, if some other condition developed first (*e.g.*, T2D), a new record was created for the patient in the analysis category of T2D only. Each new record contained an index date on the basis of development of the new condition used for calculating predevelopment and postdevelopment costs. In the example, costs before the index date (development of T2D) were assigned to the no T2D, ASCVD, or HF category in the quarters before T2D developed and to the T2D only category in the quarters after the index date (T2D onset). This process was followed for each CRM outcome and for each possible combination of CRM conditions that developed before the outcome. Unlike classical longitudinal analyses, which assesses CRMs for each patient only at baseline, this multiple record per person method allows for the inclusion of all patients who develop each CRM when other CRMs are present, even when those CRMs were not present at baseline.

The cost data were positively skewed, with a very small proportion of people affecting the upper tail of the distribution because of costly encounters or high levels of utilization. We initially modeled the cost data using a generalized linear model regression framework, specifying a gamma distribution and using a log link.^14^ The gamma model is often considered the preferred method for modeling health care costs.^15^ We compared the results of these models with models using a normal distribution with an identity link. Because of the large sample size (that was further expanded by partitioning the data into quarters), we determined that the models using the normal distribution provided results that were closer to the true average cost. Others have argued that sample means perform equally as well as transformed data and are unlikely to lead to inappropriate conclusions.^16^ For ease of interpretation, we, therefore, used the untransformed data. Given that the same individuals appear in more than one quarter, we used generalized estimating equations to model the covariance structure and used an exchangeable covariance structure. After considering univariate associations between costs and candidate independent variables (see Table 1), we adjusted costs for baseline values of age, sex, Hispanic ethnicity, BP >140/90 mm Hg, smoking, and use of antihyperglycemics, statins, and renin-angiotensin-aldosterone system inhibitors. We report adjusted mean costs over all quarters before and after onset of a new CRM. We annualized costs by multiplying by the number of quarters and dividing by four. All analyses were conducted using SAS version 9.4 (Cary, NC).

**Table 1.** Baseline characteristics of study sample

Baseline Assessment	Study Sample (*N*=387,985)
Age	50.7 (15.6)
% male	44.2%
Hispanic	6.1%
Non-Hispanic Black	2.8%
Current smoker	11.1%
Systolic BP	127 (16)
Diastolic BP	76 (10)
BMI	30 (7)
eGFR	92 (20)
Use of RAAS inhibitors	17.8%
Use of statins	14.9%
Use of antihyperglycemics	5.9%
Mean follow-up, yr	6.9 (4.6)
Median follow-up, yr (IQR)	5.9 (2.8–10.9)
Baseline CKD	4.7%
Baseline T2D	9.7%
Baseline ASCVD	8.1%
Baseline HF	2.5%

BMI, body mass index; RAAS, renin-angiotensin-aldosterone system; IQR, interquartile range; T2D, type 2 diabetes; ASCVD, atherosclerotic cardiovascular disease; HF, heart failure.

## Results

The mean age of the study sample was 50.7 (SD, 15.6) years and 44.2% were men (Table 1). The mean follow-up was 6.9 (4.6) years, and the median was 5.9 (IQR, 2.8–10.9) years. At baseline (date of first eGFR), 4.7% had CKD, 9.7% had T2D, 8.1% had ASCVD, and 2.5% had HF. Among all patients without CKD at baseline, the mean adjusted annualized costs over the 1-year period before the index date of CKD incidence were $7942 (95% confidence interval [CI], $7921 to $7962) and $18,258 ($18,157 to $18,359), or 129.9% higher after incident CKD (Table 2). Costs were higher both before and after CKD incidence when other CRM conditions were present, but the absolute annualized change in costs after CKD development was consistent, ranging from $4993 to $6644. The percentage increases ranged from 21% among patients with T2D, ASCVD, and HF to 92% among patients with none of these conditions.

**Table 2.** Adjusted annualized health care costs by cardio-renal-metabolic conditions before and after incident CKD

Comorbidity Combination		Before CKD	After CKD	Change in Costs	
No ASCVD, HF, or T2D	*No.*	270,276	20,205		
	Mean	$5602	$10,756	Numeric	$5154
	95% CI	$5598 to $5606	$10,725 to $10,787	Percentage	92.0%
ASCVD only	*No.*	29,529	8010		
	Mean	$12,247	$17,268	Numeric	$5021
	95% CI	$12,170 to $12,323	$17,115 to $17,420	Percentage	41.0%
T2D only	*No.*	45,846	7757		
	Mean	$8139	$13,299	Numeric	$5160
	95% CI	$8124 to $8155	$13,232 to $13,366	Percentage	63.4%
HF only	*No.*	5017	2167		
	Mean	$20,287	$26,931	Numeric	$6644
	95% CI	$20,029 to $20,545	$26,442 to $27,420	Percentage	32.8%
T2D+HF	*No.*	2334	1212		
	Mean	$22,664	$28,544	Numeric	$5880
	95% CI	$22,277 to $23,051	$27,894 to $29,194	Percentage	25.9%
HF+ASCVD	*No.*	7291	4087		
	Mean	$26,389	$32,392	Numeric	$6003
	95% CI	$26,107 to $26,671	$31,992 to $32,791	Percentage	22.7%
T2D+ASCVD	*No.*	12,578	4291		
	Mean	$14,242	$19,235	Numeric	$4993
	95% CI	$14,133 to $14,351	$19,032 to $19,437	Percentage	35.1%
ASCVD+T2D+HF	*No.*	5443	3280		
	Mean	$28,260	$34,084	Numeric	$5824
	95% CI	$27,958 to $28,563	$33,645 to $34,523	Percentage	20.6%
All patients	*No.*	368,802	48,115		
	Mean	$7942	$18,258	Numeric	$10,316
	95% CI	$7921 to $7962	$18,157 to $18,359	Percentage	129.9%

ASCVD, atherosclerotic cardiovascular disease; HF, heart failure; T2D, type 2 diabetes; CI, confidence interval.

Table 3 displays the mean adjusted annualized costs before and after incident T2D. Overall, costs were 84% higher after ($14,871, $14,802 to $14,940) versus before ($8082, $8059 to $8106) incident T2D. As with CKD, the cost increase after incident T2D was consistent regardless of other CRM conditions. The percentage increase was the highest among patients with no other CRM conditions (89.1%) and the lowest when all three were present (13.7%).

**Table 3.** Adjusted annualized health care costs by cardio-renal-metabolic conditions before and after incident type 2 diabetes

Comorbidity Combination		Before T2D	After T2D	Change in Costs	
No ASCVD, HF, or CKD	*No.*	276,485	51,333		
	Mean	$5600	$10,590	Numeric	$4990
	95% CI	$5596 to $5604	$10,567 to $10,613	Percentage	89.1%
ASCVD only	*No.*	28,429	9846		
	Mean	$12,281	$18,049	Numeric	$5768
	95% CI	$12,204 to $12,359	$17,874 to $18,244	Percentage	47.0%
CKD only	*No.*	15,983	4813		
	Mean	$10,379	$15,262	Numeric	$4883
	95% CI	$10,342 to $10,416	$15,134 to $15,389	Percentage	47.0%
HF only	*No.*	4618	1542		
	Mean	$20,208	$25,982	Numeric	$5774
	95% CI	$19,943 to $20,474	$25,389 to $26,576	Percentage	28.6%
CKD+HF	*No.*	2831	857		
	Mean	$24,203	$30,016	Numeric	$5813
	95% CI	$23,867 to $24,358	$29,227 to $30,804	Percentage	24.0%
HF+ASCVD	*No.*	6397	2651		
	Mean	$26,686	$31,714	Numeric	$5028
	95% CI	$26,384 to $26,988	$31,101 to $32,327	Percentage	18.8%
CKD+ASCVD	*No.*	6397	2651		
	Mean	$16,344	$21,302	Numeric	$4958
	95% CI	$16,219 to $16,469	$21,007 to $21,596	Percentage	30.3%
ASCVD+CKD + HF	*No.*	7702	2482		
	Mean	$30,600	$34,800	Numeric	$4200
	95% CI	$30,377 to $30,822	$34,246 to $35,354	Percentage	13.7%
Total	*No.*	343,559	73,615		
	Mean	$8082	$14,871	Numeric	$6789
	95% CI	$8059 to $8106	$14,802 to $14,940	Percentage	84.0%

T2D, type 2 diabetes, ASCVD, atherosclerotic cardiovascular disease; HF, heart failure; CI, confidence interval.

The mean adjusted annualized costs were four-fold greater after ($28,672, $29,544 to $29,801) compared with before ($7099, $7085 to $7114) incident ASCVD (Table 4). Although the marginal cost of incident ASCVD was not dramatically different, the percentage increases varied considerably depending on which CRM conditions were present before ASCVD. For example, costs were over two-fold (211%) higher after vs. before incident ASCVD when T2D was the only CRM present, but only 65% greater among patients with CKD and HF.

**Table 4.** Adjusted annualized health care costs by cardio-renal-metabolic conditions before and after incident atherosclerotic cardiovascular disease

Comorbidity Combination		Before ASCVD	After ASCVD	Change in Costs	
No CKD, HF, or T2D	*No.*	275,011	34,843		
	Mean	$5614	$23,093	Numeric	$17,479
	95% CI	$5610 to $5618	$22,960 to $23,226	Percentage	311.3%
CKD only	*No.*	17,633	7287		
	Mean	$10,541	$27,159	Numeric	$16,618
	95% CI	$10,507 to $10,575	$26,862 to $27,546	Percentage	157.7%
T2D only	*No.*	45,720	11,121		
	Mean	$8172	$25,426	Numeric	$17,254
	95% CI	$8156 to $8187	$25,187 to $25,666	Percentage	211.1%
HF only	*No.*	5039	5348		
	Mean	$20,692	$39,836	Numeric	$19,144
	95% CI	$20,433 to $20,952	$39,248 to $40,424	Percentage	92.5%
T2D+HF	*No.*	2262	3036		
	Mean	$22,928	$41,815	Numeric	$18,887
	95% CI	$22,543 to $23,313	$41,023 to $42,607	Percentage	82.4%
HF+CKD	*No.*	3247	3893		
	Mean	$25,017	$41,242	Numeric	$16,225
	95% CI	$24,712 to $25,323	$40,625 to $41,859	Percentage	64.9%
T2D+CKD	*No.*	8396	3709		
	Mean	$12,992	$29,929	Numeric	$16,937
	95% CI	$12,934 to $13,051	$29,492 to $30,366	Percentage	130.4%
CKD+T2D+HF	*No.*	2330	2959		
	Mean	$27,226	$45,174	Numeric	$17,948
	95% CI	$26,878 to $27,573	$44,423 to $45,925	Percentage	65.9%
Total	*No.*	354,027	68,432		
	Mean	$7099	$28,672	Numeric	$21,573
	95% CI	$7085 to $7114	$29,544 to $29,801	Percentage	303.9%

ASCVD, atherosclerotic cardiovascular disease; HF, heart failure; T2D, type 2 diabetes; CI, confidence interval.

Incident HF resulted in the greatest difference in before ($7682, $7667 to $7696) versus after ($44,204, $33,006 to $44,401) mean adjusted annualized costs or 475% higher after HF incidence (Table 5). For most CRM analysis categories, costs were at 200%–300% higher after HF, the exceptions being CKD+ASCVD (166%) and ASCVD+T2D+CKD (152%), but again, the marginal cost of incident HF was consistent, ranging from $27,296 to $29,576.

**Table 5.** Adjusted annualized health care costs by cardio-renal-metabolic conditions before and after incident heart failure

Comorbidity Combination		Before HF	After HF	Change in Costs	
No ASCVD, CKD, or T2D	*No.*	265,525	6239		
	Mean	$5607	$33,119	Numeric	$27,512
	95% CI	$5603 to $5611	$32,759 to $33,479	Percentage	490.7%
ASCVD only	*No.*	27,883	7187		
	Mean	$12,369	$41,788	Numeric	$29,419
	95% CI	$12,291 to $12,447	$41,328 to $42,249	Percentage	237.8%
T2D only	*No.*	42,599	2386		
	Mean	$8149	$35,689	Numeric	$27,540
	95% CI	$8132 to $8165	$35,096 to $36,281	Percentage	338.0%
CKD only	*No.*	16,049	2855		
	Mean	$10,494	$37,790	Numeric	$27,296
	95% CI	$10,548 to $10,530	$37,231 to $38,348	Percentage	260.1%
T2D+CKD	*No.*	7716	1644		
	Mean	$12,964	$41,326	Numeric	$28,362
	95% CI	$12,903 to $13,024	$40,562 to $42,089	Percentage	218.8%
CKD+ASCVD	*No.*	9879	5754		
	Mean	$16,772	$44,550	Numeric	$27,778
	95% CI	$16,657 to $16,887	$44,074 to $45,025	Percentage	165.6%
T2D+ASCVD	*No.*	11,729	4402		
	Mean	$14,464	$44,040	Numeric	$29,576
	95% CI	$14,352 to $14,576	$43,452 to $44,628	Percentage	204.5%
ASCVD+T2D+CKD	*No.*	6791	4510		
	Mean	$18,977	$47,805	Numeric	$28,828
	95% CI	$18,847 to $19,107	$47,240 to $48,370	Percentage	151.9%
Total	*No.*	377,075	32,435		
	Mean	$7682	$44,204	Numeric	$36,522
	95% CI	$7667 to $7696	$44,006 to $44,401	Percentage	475.4%

HF, heart failure; ASCVD, atherosclerotic cardiovascular disease; T2D, type 2 diabetes; CI, confidence interval.

## Discussion

In this time-dependent analysis of the health care costs of nearly 400,000 individuals before and after incident CKD, T2D, ASCVD, and HF, we found consistent patterns in how costs changed after the development of each CRM condition. Occurrence of any of these chronic diseases resulted in incremental cost of care regardless of which other conditions were present when the new condition developed.

Professional societies and government organizations provide national estimates of costs of T2D, CVD, HF, and CKD.^5,6,8^ However, these reports comingle the costs of each disease. For example, the $237 billion in direct medical costs of diagnosed diabetes in the United States reported by the American Diabetes Association includes patients with and without CVD, HF, and CKD.^5^ Similarly, the American Heart Association's estimates of costs for CVD include patients with T2D, HF, and CKD, and costs associated with HF include patients with T2D and CKD.^6^ Our study is novel because we estimate costs associated with developing each of the CRM conditions in the context of the presence or absence of each other condition as well as all combinations of conditions. Although absolute costs depended on the number of CRMs present, the absolute annualized change in costs after vs. before the incidence of each condition was remarkably consistent regardless of the presence of other CRM diseases alone or in combination. This finding is important because it allows health care organizations to plan for expected costs after the development of each CRM and, just as importantly, to determine the economic benefits of preventing the development of each CRM and invest in optimal management and preventive resources accordingly.

There are a number of studies reporting per-person health care costs of CRM conditions.^7,9,17–28^ Comparison with the cited studies is difficult because of differences in the systems, populations, and years analyzed as well as the year used to report costs. As noted above and to our knowledge, no studies have isolated the costs associated with each CRM condition in the context of the others, making comparisons with the current results impossible. In addition, studies of costs before and after diagnosis of any of the CRM conditions are exceedingly rare and may exist only for diabetes.^29,30^ Our novel findings are critically important to the understanding of the health care costs of CRM.

Health care costs after diagnosis of all four CRM conditions increased, but the change in costs associated with ASCVD and HF were particularly high. Treatment of these two conditions typically requires hospitalization and in the case of HF often involves costly readmissions.^31,32^ By contrast, complications of T2D (such as ASCVD and HF) may take many years to manifest after initial onset, and because CKD progression does not typically occur quickly, the extremely high costs of late-stage CKD are unlikely to be captured after CKD incidence. Our results should, therefore, be viewed in the context of the first few years after CRM incidence and not lifetime costs of each CRM. Incidence of each of the CRM conditions added an approximate constant dollar amount regardless of the presence of other conditions and thus produced a cumulative effect on total costs, emphasizing the importance of prevention of all four components of the CRM syndrome.

Strengths of our study included a time-dependent–like methodology that allowed us to isolate the costs associated with development of each CRM condition for all possible combinations of existing CRM conditions and the comprehensive capture of long-term cost data. However, the study had some potential limitations. In our data, urine-albumin creatinine ratio results were not available for patients without diabetes, so we relied solely on eGFR to define CKD, thus characterizing patients with normal eGFR but with microalbuminuria or macroalbuminuria as free of CKD. Because we would expect higher costs among patients with microalbuminuria or macroalbuminuria, including them with non-CKD patients would inflate the costs before the development of eGFR-based CKD, thereby underestimating the costs associated with developing CKD as defined in this study. We relied on diagnoses recorded in the EMR to identify CRM incidence. However, if this resulted in misassignment, costs were underestimated in the postdiagnosis periods because they included patients who had not truly developed the condition and overestimated in the prediagnosis periods because they included patients who already had the condition but had not yet been diagnosed. In either case, the result would be an underestimation of the difference between prediagnosis and postdiagnosis costs; our results are likely conservative. As an integrated delivery system, the cost structure of the study site may be unique, so readers should view absolute dollar amounts with caution, but percentage changes in costs after onset of a CRM condition should generalize to other settings. Our study sample included only patients who had a serum creatinine test result. Although this a common test and part of a comprehensive metabolic panel, we by definition excluded healthy patients who had not sought medical care. We may also have missed patients with CKD who had a low eGFR but no confirmatory value available or in case two eGFR values <60 ml/min per 1.73 m^2^ were more than 12 months apart. As a result, we may be overestimating prediagnosis costs, again resulting in conservative estimates of the change in costs after diagnosis. Other limitations inherent in all observational studies, such as nonrandomization and inability to account for unmeasured variables, are also present. Specifically, we did not account for costs associated with comorbidities other than the included CRMs.

In conclusion, we found that development of CKD, T2D, ASCVD, and HF drove substantial health care costs. The *absolute* dollar amounts associated with the incidence of each of these CRM conditions was approximately constant regardless of whether other conditions were already present. As a result, and not surprisingly, the cost effects of CRM conditions were cumulative. Our findings indicate a clear interplay of CRM condition that drive substantial health care costs and emphasize the common economic need for prevention of CKD, T2D, ASCVD, and HF.
